# Supplementary material for: Transcriptomic and biochemical insights into fall armyworm (Spodoptera frugiperda) responses on silicon-treated maize
Source: PeerJ. 2024 Feb 23;12:e16859. doi: 10.7717/peerj.16859 (PMC10896081; doi:10.7717/peerj.16859)
Supplement: Supplemental Information 1 [file peerj-12-16859-s001.docx]

**Table S1**

The primers used in this study.

| **Table S1.** List of forward and reverse primers used in qPCR | | | | |
| --- | --- | --- | --- | --- |
| **Genes** | **NR ID** | **Forward primer** | **Reverse primer** |  |
| LOC118270266 | XP_039305031 | AGGGCCGAGCACATCAGGTA | GCATGCTCTCCCACTCGTCC |  |
| LOC118274873 | XP_011170020 | CCGACATCGCCCTGATCCAC | TACCTCACGGCCCAGTCGAA |  |
| LOC118270630 | XP_011157846 | TGGTGGTGTTCCTGCTGCTG | GGATGGCGGGCAGGTACTTC |  |
| LOC118279657 | XP_025987215 | GTGACCGTGGTGGTGCTGAA | CTGCCTGCTGTCGTAGTCGG |  |
| LOC118270269 | XP_025993146 | AGGGGCATCATGGAGGTGGT | CTTGCCGTCCTTGCTGTCCA |  |
| LOC118273370 | XP_011165663 | CCTTCAAGGACGGCACCCTG | GGGGGCCACTTGCTGATCTC |  |
| LOC118274553 | XP_011164602 | GCAGGATCAGGGGCACCATC | TCACCCTCAGGCAGTCCTCC |  |
| LOC118279656 | XP_039306574 | GAACCACACCGGCAAGGTGA | AGGATGTCCCACTCCACGCT |  |
| LOC118270522 | XP_039303557 | ACCACAGGGTGCACCACAAG | TCCCAGAACACCACGGGGAT |  |
| LOC118275355 | XP_025993436 | GCACCCCGACGTGAAGAACA | AGGCCCAGGCAGTACCTGAA |  |
| LOC118269824 | XM_011167343 | TCCCCTTTACGGCATTGGGC | CGGGAAGCTGCGGACTGATT |  |
| LOC118276076 | XP_025993436 | GCACCCCGACGTGAAGAACA | AGGCCCAGGCAGTACCTGAA |  |
| LOC118273407 | XP_011165663 | CTTCAAGGACGGCACCCTGG | GGGGGCCACTTGCTGATCTC |  |
| LOC118262510 | XP_025988401 | CAAGGCCAAGTGGCCCATGA | ACGGGGTCCTTCTCCAGGTC |  |
| LOC118274789 | XP_025994943 | GATCGAGCAGGACGTGGTGG | CTCGCCGAAGATGGTCACCC |  |
| LOC118282340 | XP_039305287 | ACAACGCCAAGAGGGGCTTC | CCAGCTCGGCGGTCTTGTAG |  |
| LOC118266761 | XP_025992124 | CATCGAGCTGACCAACGGCT | TCCTGTCCAGGTCCCTCTGC |  |
| LOC118275358 | XP_039305031 | AGGGCCGAGCACATCAGGTA | AGGGCCGAGCACATCAGGTA |  |
| LOC118279296 | XP_011157846 | TGGTGGTGTTCCTGCTGCTG | GGATGGCGGGCAGGTACTTC |  |
| LOC118275193 | XP_011165663 | CCTTCAAGGACGGCACCCTG | GGGGGCCACTTGCTGATCTC |  |

|  |
| --- |
|  |
|  |
|  |
|  |
|  |
|  |
|  |
|  |
|  |
